# Implementation of patient dosimetry in the clinical practice after targeted radiotherapy using [^177^Lu-[DOTA0, Tyr3]-octreotate

**DOI:** 10.1186/s13550-018-0459-4

**Published:** 2018-11-29

**Authors:** Lore Santoro, Erick Mora-Ramirez, Dorian Trauchessec, Soufiane Chouaf, Pierre Eustache, Jean-Pierre Pouget, Pierre-Olivier Kotzki, Manuel Bardiès, Emmanuel Deshayes

**Affiliations:** 1 Nuclear Medicine Department, Montpellier Cancer Institute (ICM), University of Montpellier, 208 Avenue des Apothicaires, 34298 Montpellier Cedex5, France; 2 Centre de Recherche en Cancérologie de Toulouse, Toulouse, France; 3 INSERM, UMR 1037, Toulouse III Paul Sabatier University, Toulouse, France; 4 University of Costa Rica, Physics School, CICANUM, San Jose, Costa Rica; 5 Institut de Recherche en Cancérologie de Montpellier (IRCM), INSERM U1194, Université de Montpellier, Institut Régional du Cancer de Montpellier (ICM), Montpellier, France

**Keywords:** Peptide receptor radionuclide therapy, [^177^Lu- [DOTA0, Tyr3]-octreotate, Medical internal radiation dose, Patient-specific dosimetry

## Abstract

**Background:**

This study’s aim was to develop our dosimetric methodology using a commercial workstation for the routine evaluation of the organs at risk during peptide receptor radionuclide therapy (PRRT) with ^177^Lu.

**Methods:**

First, planar and SPECT sensitivity factors were determined on phantoms. The reconstruction parameters were optimized by SPECT/CT image acquisition using a NEMA IEC phantom containing a 500 ml bottle of ^177^Lu, to simulate a kidney. The recovery coefficients were determined on various phantoms. For the red marrow, this was calculated using a NEMA IEC phantom that contained a centrally placed bottle of 80 ml of ^177^Lu (to model the L2-L4 red marrow) flanked by two 200 ml bottles with ^177^Lu to simulate the kidneys.

Then, SPECT/CT images were acquired at 4, 24, 72, and 192 h after injection in 12 patients with neuroendocrine tumors who underwent PRRT with ^177^Lu-DOTATATE. SPECT data were reconstructed using the iterative ordered subset expectation maximization (OSEM) method, with six iterations and ten subsets, attenuation, scatter, recovery resolution corrections, and a Gaussian post-filter of 0.11 cm. The liver, spleen, kidneys, and red marrow dose per administered activity (AD/A admin) values were calculated with the Medical Internal Radiation Dose (MIRD) formalism and the residence times (Dosimetry toolkit® application) using standard and CT imaging-based organ masses (OLINDA/EXM® V1.0 software).

**Results:**

Sensitivity factors of 6.11 ± 0.01 and 5.67 ± 0.08 counts/s/MBq were obtained with planar and SPECT/CT acquisitions, respectively. A recovery coefficient of 0.78 was obtained for the modeled L2–L4 red marrow. The mean AD/A admin values were 0.43 ± 0.13 mGy/MBq [0.27–0.91] for kidneys, 0.54 ± 0.58 mGy/MBq [0.12–2.26] for liver, 0.61 ± 0.13 mGy/MBq [0.42–0.89] for spleen, and 0.04 ± 0.02 mGy/MBq [0.01–0.09] for red marrow. The AD/A admin values varied when calculated using the personalized and standard organ mass, particularly for kidneys (*p* = 1 × 10^−7^), spleen (*p* = 0.0069), and red marrow (*p* = 0.0027). Intra-patient differences were observed especially in organs close to or including tumor cells or metastases.

**Conclusions:**

The obtained AD/A admin values were in agreement with the literature data. This study shows the technical feasibility of patient dosimetry in clinical practice and the need to obtain patient-specific information.

## Background

In recent years, new radiopharmaceuticals, such as radiopeptides, have been developed for targeted radiotherapy. Consequently, in addition to red marrow, healthy liver and kidneys also are now identified as organs at risk (OAR) [1, 2]. Moreover, the increasing evidence that treatment outcomes correlate with the absorbed doses delivered to tumors and healthy organs [3, 4] makes individualized dosimetry all the more necessary [5].

Peptide receptor radionuclide therapy (PRRT) is a promising treatment for patients with neuroendocrine tumors (NET). Recently, the NETTER-1 trial [6] showed that treatment with ^177^Lu-DOTATATE results in markedly longer progression-free survival and higher response rate than treatment with high-dose octreotide alone in patients with advanced midgut NET. In September 2017, ^177^Lu-DOTATATE (LUTATHERA®) was approved for this indication by the European Medicines Agency. It has been shown that ^177^Lu-DOTATATE uptake in kidneys and tumors greatly varies among patients [7], and bone marrow and kidneys are considered as dose-limiting organs. Although no clear cumulative absorbed dose cut-off has been identified in PRRT with ^177^Lu to predict the risk of organ failure, which is probably multifactorial [4, 8], 23 Gy for kidneys and 2 Gy for bone marrow are sometimes proposed, although these values are based on studies with fractionated external beam radiotherapy (EBRT) that has different physical and radiobiological mechanisms. For example, based on fractionated EBRT, a 5% risk of renal dysfunction at 5 years has been described for a mean absorbed dose of 18–23 Gy and 0.5–1.25 Gy/fraction [9, 10].

In PRRT with ^177^Lu, the important inter-patient variations in peptide pharmacokinetics require treatment individualization by tailoring the number of cycles or the administered activity [11]. In this context, therapy planning based on the maximum tolerable absorbed dose to non-target organs (“as high as safely attainable”, AHASA approach) [12] could be considered, instead of the “as low as reasonably achievable” (ALARA) approach (i.e., the dose to non-target tissues should be reasonably low). However, currently, a fixed activity of 7.4 GBq per cycle, as described in the NETTER-1 trial, is usually administered. Hence, personalized dosimetry is often performed mainly to ensure safety and evaluate the absorbed dose to the tumor rather than to optimize the administered activity and to assess the dose-response relationship.

Patient dosimetry requires the accurate estimation of the activity in the targeted organs at several time points [13, 14]. Therefore, the preliminary calibration and quantification steps are crucial [15, 16]. Although there are Medical Internal Radiation Dose (MIRD) guidelines [17, 18], a standardized dosimetry protocol to evaluate safety and toxicity, and to perform dosimetric evaluations is crucially required. The need to determine the absorbed doses delivered to kidneys after ^177^Lu-DOTATATE treatment was an evidence for nuclear medicine physicians in our department [11]. Therefore, before starting the first treatment with ^177^Lu-DOTATATE, we implemented the imaging protocol based on the MIRD pamphlet n°26 [18], and performed a preliminary study on phantoms. Then, we defined the imaging schedule for patient dosimetry based on published data and our own institute logistics. We performed the dosimetry analyses using the MIRD formalism and the tools available in our nuclear medicine department. Using this protocol for dosimetry after ^177^Lu-DOTATATE treatment and a commercial workstation, we could calculate the absorbed doses per unit of administered activity (AD/A admin) in the OARs (kidneys, liver, spleen, and red marrow) in patients with NET.

## Material and methods

### SPECT/CT equipment

All imaging acquisitions were performed using the SPECT/CT Discovery NM/CT 670 system (General Electric [GE] Healthcare) with a Bright Speed 16 CT scanner and 3/8-in NaI(Tl) crystal thickness. Nuclear medicine images were acquired using a medium-energy general purpose parallel-hole collimator. A 20% energy window centered on the 208 keV photopeak and a 10% scatter correction window centered on 177 keV were applied [18]. A 128 × 128 pixel matrix was used.

### Software

The Dosimetry Toolkit®, an application of the Xeleris® software provided with the SPECT/CT Discovery NM/CT 670 system (GE Healthcare) [19], was used to determine the radiopharmaceutical residence time in segmented organs with the multi-SPECT/CT scenario, as described by Kupitz et al. [20]. First, the application “Preparation for dosimetry toolkit” was used for SPECT/CT raw data reconstruction and CT data registration. Then, the “Dosimetry Toolkit” application was used to segment the different organs, to create the time activity curves fitted by a mono-exponential function, and to calculate the residence time.

The OLINDA/EXM® V1.0 software [21] was used to calculate the organ absorbed and effective doses. It contains a large pre-established database of radionuclides and S factors to calculate the absorbed dose per unit of administered activity. Standard or patient-adapted organ masses can be used with this software. The residence times obtained for each organ with Dosimetry Toolkit® were used as input data.

### Preliminary study on phantoms

#### Correction maps

Energy and uniformity correction maps were computed using static data, acquired without collimator, of a syringe containing 91.8 MBq of ^177^Lu placed at a distance that corresponded to five times the field of view. A 10% energy window centered on the 208 keV photopeak was applied. A 256 × 256 pixel matrix and 10,000 kcounts were used for the energy and uniformity correction maps, and a 512 × 512 pixel matrix and 60,000 kcounts for the uniformity correction map.

#### Sensitivity factors

The system sensitivity was evaluated with two methods (see below). The sensitivity factor (expressed in counts/s/MBq) allowed converting the numbers of events (counts) detected by the gamma camera into activity values. For each method, the used activity was accurately measured with a CRC-25R from Capintec dose calibrator (Berthold Technologies). The calibration factor was calculated using a calibration vial of ^177^Lu (Advanced Accelerator Applications, Saint Genis Pouilly, France), with a maximum activity measurement error of 5%. The clock was synchronized with the gamma camera clock to allow accurate decay correction using a half-life of 6.647 days.

##### Method 1 (planar sensitivity factor)

According to the manufacturer’s recommendations (GE Healthcare), a syringe of ^177^Lu containing an activity of 59.1 ± 2.9 MBq was placed on the examination table of the gamma camera at 10 cm from the detectors. Planar imaging was performed for 5 min to obtain the number of events detected by the detectors. The geometric mean was calculated and reported to the real activity within the syringe and the acquisition time.

Then, a 16 ml hollow sphere filled with ^177^Lu (75.8 ± 3.8 MBq activity) was placed in the air between the detectors. Planar acquisitions were performed at different distances from the detectors (8, 13, and 18 cm) for 5 min to determine the system sensitivity.

##### Method 2 (SPECT sensitivity factor)

A NEMA IEC phantom (Body Phantom NU2-2001/2007) was filled with non-radioactive water. A 500 ml bottle to simulate a kidney was filled with 0.54 ± 0.03 MBq/ml of ^177^Lu solution and fixed inside the phantom. SPECT/CT images were acquired using the body contour option, rotation of 180° per detector, total of 60 projections, and 120 s/each. For attenuation correction, CT images were acquired (120 kV, 200 mA, slice thickness: 1.25 mm, rotation time: 0.8 s, pitch: 1.375, 512 × 512 pixel matrix), with standard reconstruction. For SPECT acquisitions, the ordered subset expectation maximization (OSEM) iterative reconstruction algorithm was used, with optimized reconstruction parameters.

#### Optimization of the reconstruction parameters

The reconstruction parameters were optimized using the SPECT/CT images acquired with the previously used NEMA IEC phantom. Using the “Preparation for Dosimetry toolkit” application, SPECT data were reconstructed with different numbers of iterations and subsets, with or without the manufacturer’s corrections (scatter, CT-based attenuation correction, resolution recovery), and different Gaussian post-filters. With the “Dosimetry toolkit” application, regions of interest were segmented in the CT slices. Information about the radionuclide and the previously computed planar sensitivity factor were entered in the appropriate interface. The segmented volume and internal activity were obtained for each evaluated reconstruction, and compared with those expected for the phantom.

#### Recovery coefficients

The recovery coefficients (i.e., the ratio between the activity concentration estimated from the image and the true activity concentration in the object) [17] were evaluated using two SPECT/CT acquisitions performed using a Deluxe Jaszczak phantom. For both acquisitions, six hollow spheres of 0.5 ml, 1.0 ml, 2.0 ml, 4.0 ml, 8.0 ml, and 16.0 ml, containing 0.537 ± 0.028 MBq/ml of ^177^Lu, were placed inside the phantom. For the first acquisition, the phantom was filled with non-radioactive water, and for the second one, with 474.3 ± 23.7 MBq of ^177^Lu (i.e., a background concentration of 0.074 ± 0.004 MBq/ml). The SPECT/CT acquisition parameters were the same as those used with the NEMA IEC phantom.

The recovery coefficient of a large volume (500 ml) was estimated by SPECT/CT using the previously described NEMA IEC phantom.

A second NEMA IEC phantom (Body Phantom NU2-2001/2007) that contained a central bottle of 80 ml with 2.2 ± 0.11 MBq of ^177^Lu (3.5 cm × 3.5 cm × 6.5 cm; to model the red marrow at the L2–L4 level) flanked by two 200 ml bottles (with 73.7 ± 3.7 MBq of ^177^Lu/each, 30-fold higher activity to simulate the kidneys) was also used. The background was filled with non-radioactive water. SPECT/CT images were acquired using the body contour option, rotation of 180° per detector, 60 projections, and 45 s/each. For attenuation correction, CT images were acquired (120 kV, automatic mA regulation with a max at 200 mA, a noise index at 6.43, slice thickness of 5 mm, rotation time of 0.8 s, pitch 1.375, 512 × 512 pixels matrix), with standard reconstruction.

For all SPECT acquisitions, the OSEM iterative reconstruction method algorithm was used with the reconstruction parameters selected after optimization (see previous section).

### Clinical dosimetry method application

#### Patients and treatment

Between May 2016 and February 2018, 12 patients (10 men and 2 women) with a NET who underwent PRRT with ^177^Lu-[DOTA0, Tyr3]-octreotate (LUTATHERA®; Advanced Accelerator Applications, Saint Genis Pouilly, France) were included in this dosimetric evaluation. Table 1 shows the patients’ characteristics. The study was approved by the local ethics review board.

**Table 1:** Patients’ characteristics

Patient	Sex	Age (years)	Weight (kg)	Primary tumor	Metastases	Injected activity (MBq)		Number of treatment cycles	SPECT/CT acquisition times	
						*C*1	*C*2		*C*1	*C*2
1	M	75	74	Small intestine NET	Nodes, mesentery, liver	7167	7073	4	NA	4 h, 24 h, 72 h, 192 h
2	M	59	72	Small intestine NET	Nodes, liver, bone	7287	†	1	4 h, 24 h, 168 h	†
3	M	82	70	Small intestine NET	Nodes, mesentery, bone	7180	6642	2	4 h, 24 h, 72 h	4 h, 24 h, 72 h, 192 h
4	M	71	71	Pancreas NET	Liver	7054	7134	4	4 h, 24 h, 72 h, 192 h	4 h, 24 h, 192 h
5	F	63	56	Pancreas NET	Liver	7323	7071	4	4 h, 24 h, 72 h, 192 h	4 h, 24 h, 72 h
6	M	59	79	Small intestine NET	Mesentery, liver	7207	7188	4	4 h, 24 h, 72 h, 192 h	4 h, 24 h, 72 h, 192 h
7	F	82	57	Pancreas NET	Liver	7177	7239	4	4 h, 24 h, 72 h, 192 h	4 h, 24 h, 72 h, 192 h
8	M	61	74	Small intestine NET	Nodes, mesentery, liver, bone	7298	7210	4	4 h, 24 h, 72 h, 192 h	4 h, 24 h, 72 h, 192 h
9	M	70	88	Small intestine NET	Liver, bone	7222	7158	4	4 h, 24 h, 192 h	4 h, 24 h, 72 h, 192 h
10	M	78	89	Large intestine NET	Nodes, liver	7162	6620	2	4 h, 24 h, 72 h, 192 h	4 h, 24 h, 192 h
11	M	53	70	Small intestine NET	Liver, bone	7102	7260	3	4 h, 24 h, 72 h, 192 h	4 h, 24 h, 72 h, 192 h
12	M	74	70	Small intestine NET	Nodes, mesentery	7288	7578	3	4 h, 24 h, 72 h, 192 h	4 h, 24 h, 72 h, 192 h

*NA* not available, *C* cycle, *NET* neuroendocrine tumor, † patient dead

PRRT consisted in one intravenous injection of 7.4 GBq LUTATHERA® every 8 weeks for a total of four cycles. Lysine and arginine were administered concomitantly to ensure kidney protection by reducing the tubular reabsorption of the radiolabeled peptides. All patients were hospitalized in special radioprotection rooms for 24 h after injection. After injection, the residual activity in the vial was measured with the Capintec CRC-25R dose calibrator. By taking into account the ^177^Lu physical decay, the real administered activity was determined by subtracting the residual activity in the vial from the activity before injection.

#### Dosimetry imaging protocol

Dosimetry calculations were based on the imaging data collected after the first two cycles. Images were acquired with the SPECT/CT Discovery NM/CT 670, at 4 h, 24 h, 72 h, and 192 h after the first and second injection. In total, 60 projections (45 s per projection) were acquired with a 128 × 128 pixel matrix (pixel size: 4.416 mm). For medical consideration, a whole body (WB) scan was performed at 72 h with a scan speed of 15 cm/min. At 4 h post-injection, CT images were acquired with a better image quality (120 kV, automatic mA regulation with max = 200 mA, noise index of 6.43, slice thickness of 5 mm, rotation time of 0.8 s, pitch 1.375, 512 × 512 pixel matrix, and standard reconstruction filter). For this first CT scan, patient set-up and immobilization devices were recorded to reproducibly position the patients for the next SPECT/CT image acquisitions. The other CT scans were acquired with the same parameters, except for the rotation time (0.6 s) and 80 mA fixed. After the third and fourth cycles, SPECT/CT and WB scan were performed only at 24 h.

#### Dosimetry calculations

Following the last SPECT/CT acquisition, at 192 h after cycle 2, all SPECT/CT data were loaded on the “Preparation for dosimetry toolkit” application. Transversal slices were reconstructed using the OSEM algorithm with the reconstruction settings defined in the optimization study associated with correction of the patient’s movements. Each SPECT acquisition was registered with the corresponding CT acquisition. A rigid registration between CT scans was performed. The reconstruction results were loaded on the “Dosimetry toolkit” application. The OARs (red marrow, kidneys, liver, and spleen) were segmented using the CT and SPECT images collected at 4 h post-injection. They were then replicated for the 24 h, 72 h, and 192 h images. The red marrow absorbed dose was determined by delineating the trabecular section on the L2–L4 lumbar vertebrae, considering that this section represents 6.7% of the total red marrow [22]. For kidneys, the delineation encompassed the cortex and the medulla regions of the left and right kidney, as described by Sundlöv et al. [11]. For the kidney volume, the partial volume effect correction was considered negligible. For the trabecular section on the L2–L4 lumbar vertebrae, the obtained recovery coefficient was applied.

The residence times were calculated with the SPECT sensitivity factor, and then exported to OLINDA/EXM® V1.0 to calculate the AD/A admin values using the standard organ masses included in the software and the personalized organ masses. These were determined using the volume of each organ of interest defined on the CT images and the biological tissue density proposed by the Monte Carlo Gate database and by Vieira et al. [23]*.*

The relative difference (in %) between the personalized and standard mass for organ *i* and patient *j* (Δ_mass *i*, *j*_) was defined as follows:

$$ {\Delta}_{\mathrm{mass}\ i,j}=100\times \frac{\mathrm{Personalized}\ {\mathrm{organ}\ \mathrm{mass}}_{\mathrm{ij}}-\mathrm{Standard}\ {\mathrm{organ}\ \mathrm{mass}}_{ij}}{\mathrm{Standard}\ {\mathrm{organ}\ \mathrm{mass}}_{\mathrm{ij}}} $$

where the personalized organ mass_*ij*_ is the mass of organ *i* for patient *j* calculated from the CT images and the biological tissue density, and the standard organ mass_*ij*_ is the mass of organ *i* for patient *j* included in the OLINDA/EXM®V1.0 software.

The AD/A admin values obtained with personalized and standard organ masses for each patient (calculated after cycle 1 and cycle 2) were compared with the paired Student’s *t* test (*n* = 22). To study the intra-patient variability, the personalized absorbed doses after cycle 1 and 2 (*n* = 10) were compared using the paired Student’s *t* test.

The difference (in %), between the organ *i* AD/A admin after cycle 1 and 2 and for the patient *j* (Δ_AD/Aadmin i,j_), was defined as follows:

$$ {\Delta}_{AD/\mathrm{A}\ \mathrm{admin}\  ij}=100\times \frac{SD\ \left(\mathrm{AD}/\mathrm{A}\ {\mathrm{admin}}_{1 ij,}\mathrm{AD}/\mathrm{A}\ {\mathrm{admin}}_{2 ij}\right)}{\mathrm{Arithmetic}\ \mathrm{mean}\ \left(\mathrm{AD}/\mathrm{A}\ {\mathrm{admin}}_{1 ij,}\mathrm{AD}/\mathrm{A}\ {\mathrm{admin}}_{2 ij}\right)} $$

where SD is the standard deviation, and AD/A admin _1*ij*_ and AD/A admin _2*ij*_ are the organ i AD/A admin for patient j after cycle 1 and cycle 2, respectively.

${\overline{\Delta}}_{AD/\mathrm{A}\ \mathrm{admin}\ i}$ (in %) represents the mean difference of the organ *i* AD/A admin values for all patients (*j* = 1 to *j* = 10) and was calculated as follows:

$$ {\overline{\Delta}}_{AD/\mathrm{A}\ \mathrm{admin}\ i}=\frac{1}{j}\sum \limits_{j=1}^{j=10}{\Delta}_{AD\  ij} $$

## Results

### Preliminary imaging optimization/calibration with phantoms

**Fig. 1:**
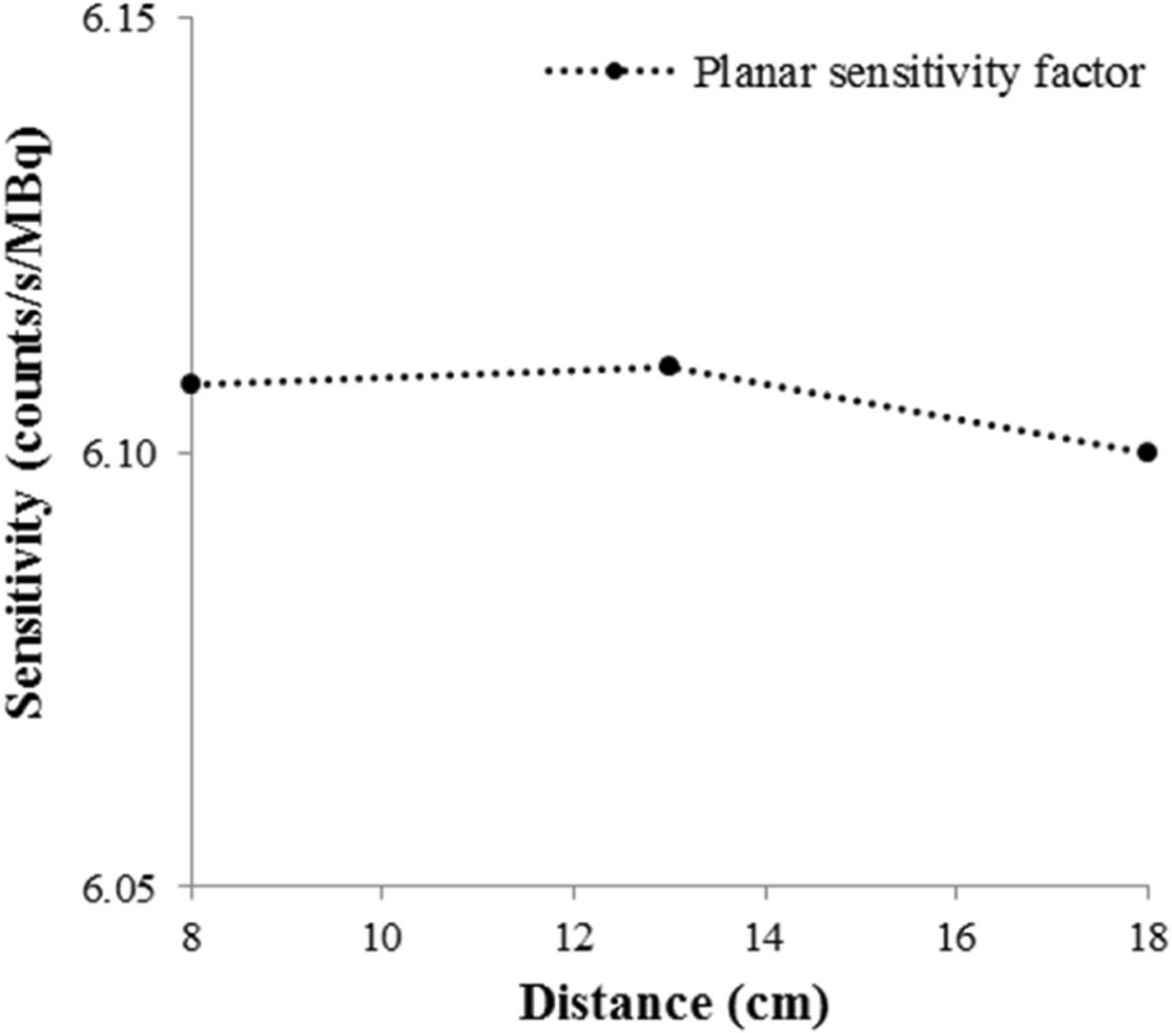
Planar sensitivity variation in function of the distance from the detectors of the gamma camera GE-Discovery NM/CT 670. For each distance (8 cm, 13 cm, 18 cm), the planar sensitivity was estimated from the geometric mean using the Xeleris® software. A circular region of interest that delineates the source distribution was used. The planar sensitivity factor remained constant (6.11 ± 0.01 counts/s/MBq) from 8 to 18 cm

The obtained planar sensitivity factor of 6.11 ± 0.01 counts/s/MBq did not vary significantly with the distance from the detectors (Fig. 1) and was then used to evaluate the optimized reconstruction parameters. The OSEM algorithm with six iterations and ten subsets and including all the corrections, associated with a Gaussian post-filter of 0.11 cm, gave the most accurate activity quantification with a relative difference of − 5.2% from the expected concentration activity in the phantom (Table 2).

**Table 2:** Deviation between the activity concentration calculated from SPECT/CT images and the real activity concentration inside the bottle to simulate a kidney

OSEM reconstruction corrections	Number of iterations (i) and subsets (ss)	Filter	Calculated activity concentration (MBq/ml)	Real activity concentration (MBq/ml)	Deviation (%)
AC + RR + SC	4i*5ss	None	0.506	0.537	− 5.88
	4i*10ss		0.495		− 7.88
	6i*10ss		0.502		− 6.55
	8i*10ss		0.503		− 6.33
	16i*10ss		0.502		− 6.55
	32i*10ss		0.507		− 5.66
	4i*5ss	Gaussian 0.11 cm	0.507		− 5.66
	4i*10ss		0.494		− 88.0
	6i*10ss		0.509		− 5.22
	8i*10ss		0.507		− 5.66
	16i*10ss		0.505		− 66.0

*AC* attenuation correction, *RR* recovery resolution, *SC* scatter correction

A SPECT/CT sensitivity factor of 5.67 ± 0.08 counts/s/MBq was obtained with these optimized reconstruction parameters.

**Fig. 2:**
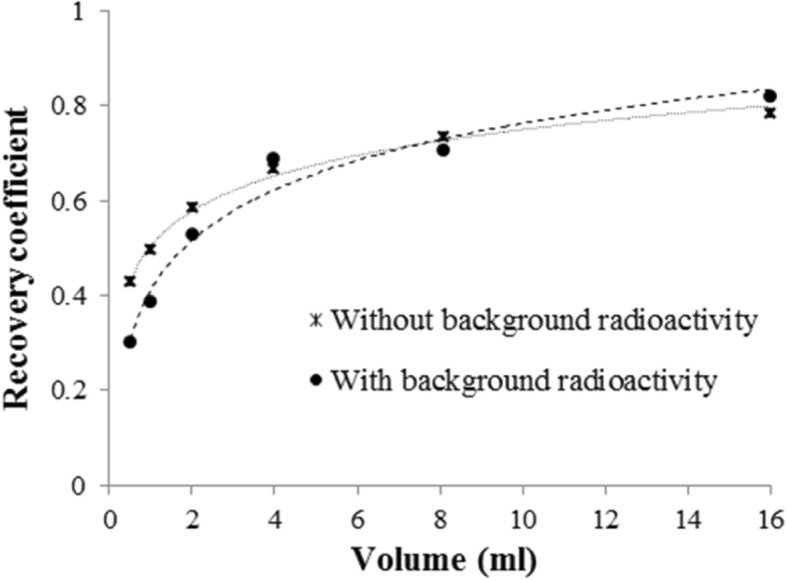
Recovery coefficients in function of the volume using six iterations and ten subsets with a Gaussian post-filter of 0.11 cm, and attenuation, scatter and recovery resolution correction. For the objects ranging from 0.5 to 16 ml, the recovery coefficients ranged from 0.43 to 0.78 when using the phantom without background radioactivity (dash-dot line) and from 0.30 to 0.82 with the phantom with background radioactivity (dotted line)

The recovery coefficient obtained for the SPECT/CT acquisition of a 500 ml volume of ^177^Lu was 0.95. For smaller spheres (from 0.5 to 16 ml), the recovery coefficients were significantly lower, ranging from 0.43 to 0.78 for the phantom without background activity, and from 0.30 to 0.82 for the phantom with background activity (Fig. 2).

A recovery coefficient of 0.78 was obtained for the phantom with the central 80 ml bottle (L2–L4 red marrow model) flanked by two 200 ml bottles to simulate the kidneys.

### Patient dosimetry

#### Patients

Dosimetry was performed after the first two treatment cycles, but for patients 1 and 2. Patient 1 did not have evaluable SPECT/CT data after cycle 1, and patient 2 died before the second cycle of ^177^Lu- [DOTA0, Tyr3]-octreotate. Patients received between two and four cycles of ^177^Lu-[DOTA0, Tyr3]-octreotate (Table 1). For six patients, SPECT/CT image acquisitions were not performed at all four time points after the first and second cycle (Table 1), mainly due to health reasons, technical issues, or logistic reasons.

#### Dosimetry

**Fig. 3:**
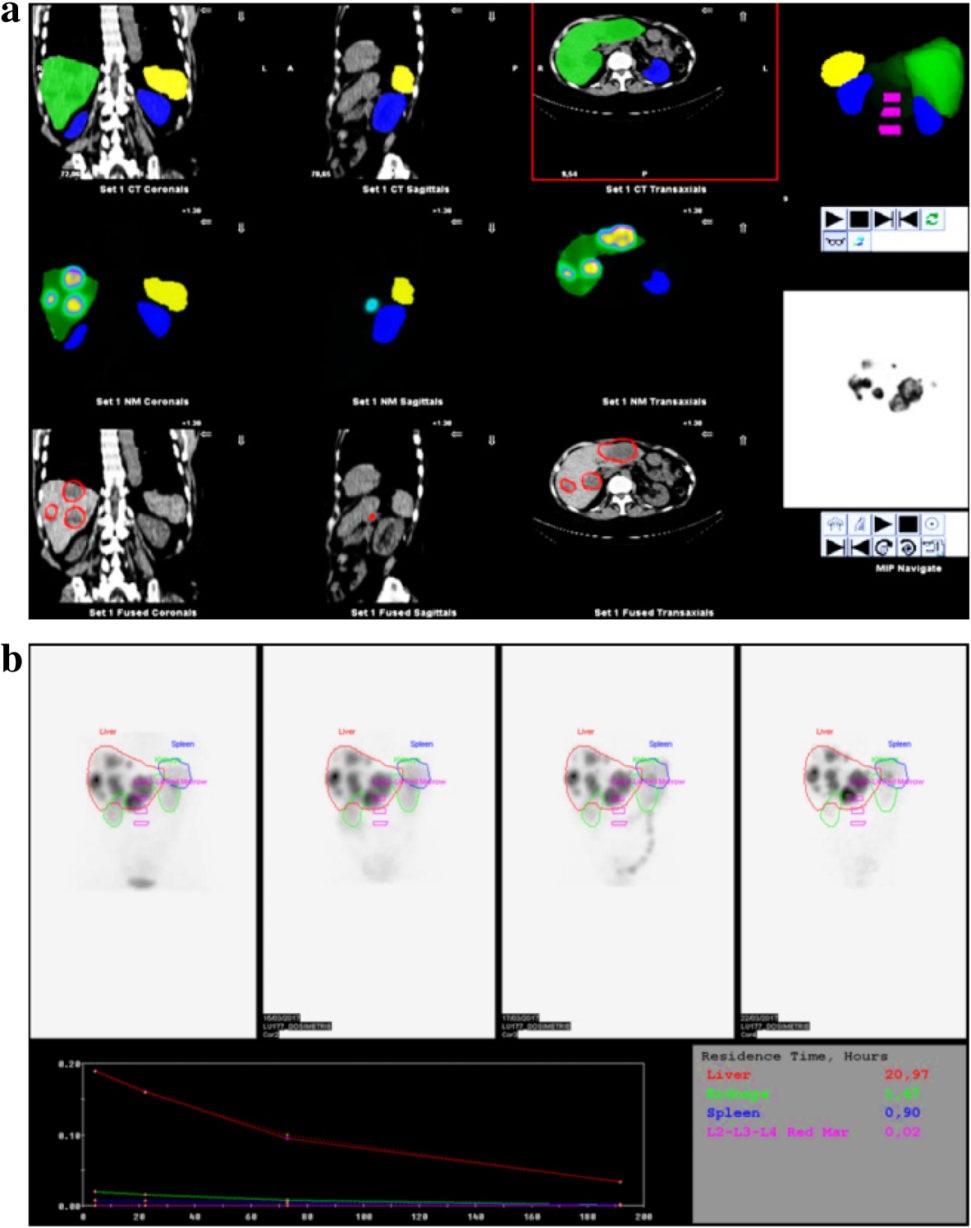
Dosimetry toolkit® workflow. **a** Example of segmentation of the organs at risk performed manually using the Dosimetry Toolkit® tools using SPECT or CT data. **b** Segmentation propagation, time activity curves and residence times for all the regions of interest at 4 h, 24 h, 72 h and 192 h after drug administration

Time activity curves and residence times (*n* = 12 patients) were computed for liver, kidneys, spleen, and red marrow (Fig. 3).

##### Personalized organ mass versus standard organ mass

The mean personalized organ masses were 1962 g for liver, 470 g for kidneys, 296 g for spleen, and 891 g for red marrow. The maximum Δ_mass *i*,*j*_ between personalized and standard mass was 130.3% for liver (patient 2) and 307.2% for spleen (patient 11). The Δ_mass *i*,*j*_ ranged from 0.8 to 93.5% for kidneys, and from − 55.1 to 11.9% for red marrow.

**Fig. 4:**
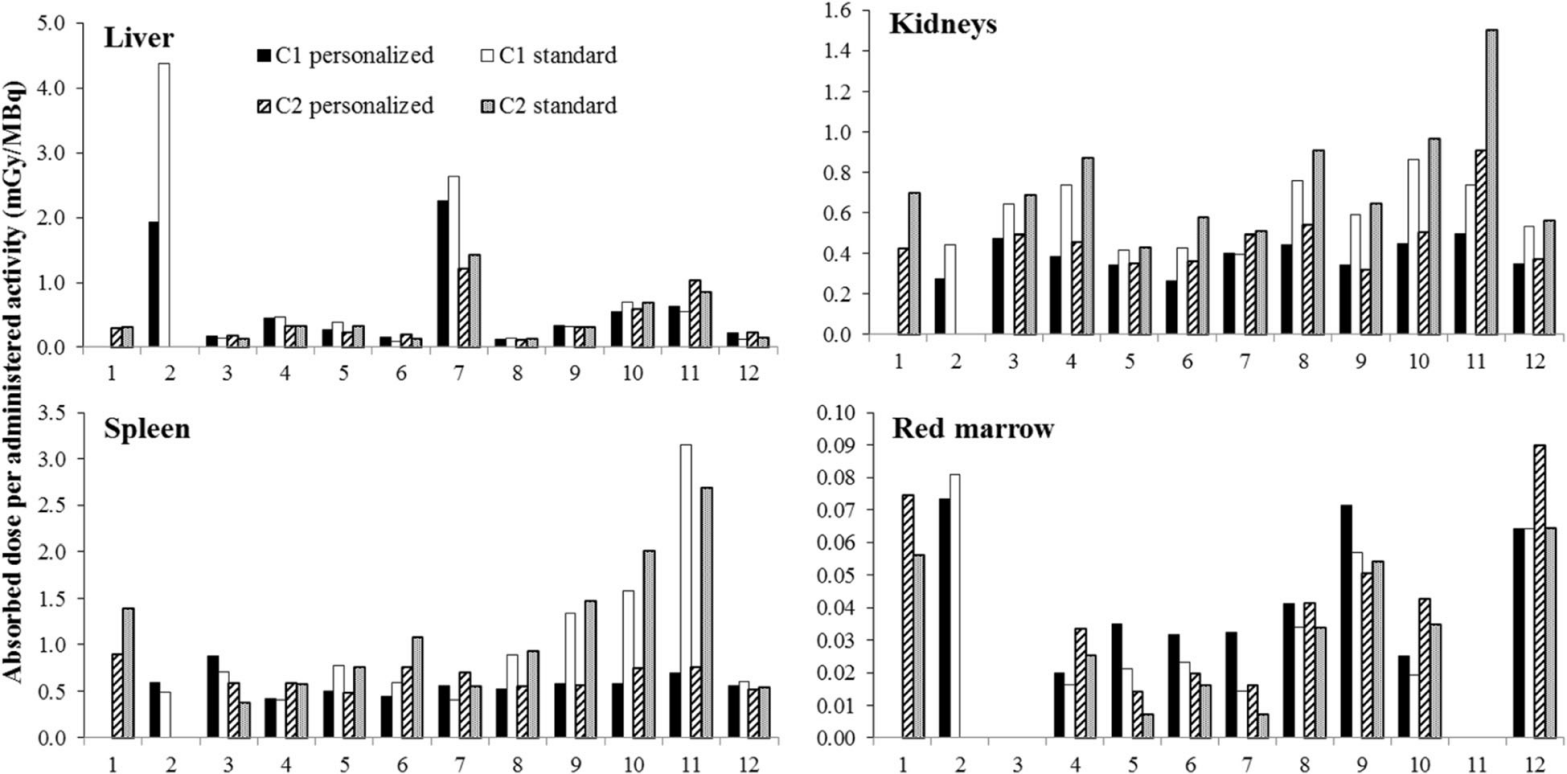
Absorbed dose per administered activity (in mGy/MBq) calculated with personalized (CT scan-based) organ masses and standard organ masses (given by OLINDA/EXM® V1.0 software) after cycle 1 (C1 personalized and C1 standard) and cycle 2 (C2 personalized and C2 standard). As our dosimetry methodology was based on L2-L3-L4 red marrow delineation, it could not be used for patients 3 and 11 who had tumors in contact with L2, L3, L4 and L2, L3, or L4 vertebral metastases

In agreement, the AD/A admin values for the four OARs varied when calculated using the personalized and standard organ mass, particularly for kidneys (*p* = 1 × 10^−7^), spleen (*p* = 0.0069), and red marrow (*p* = 0.0027) (Fig. 4). For example, the kidney AD/A admin values for patient 11 after cycle 2 were 0.91 mGy/MBq with the personalized and 1.50 mGy/MBq with the standard organ mass. In patient 12, the red marrow AD/A admin values after cycle 2 reached a value of 0.09 mGy/MBq with the personalized organ mass and 0.06 mGy/MBq with the standard one. A large difference was observed for the spleen AD/A admin values, especially in patient 11 after cycle 1 (0.70 mGy/MBq versus 3.15 mGy/MBq).

##### AD/A admin values after cycle 1 and 2

The AD/A admin values obtained using the personalized organ masses are presented in Table 3. The highest AD/A admin values were 2.26 mGy/MBq in liver (patient 7 after cycle 1), 0.91 mGy/MBq in kidneys (patient 11 after cycle 2), 0.88 mGy/MBq in spleen (patient 3 after cycle 1), and 0.09 mGy/MBq in red marrow (patient 12 after cycle 2).

**Table 3:** Absorbed dose per administered activity (AD/A admin; in mGy/MBq) to the liver, kidneys, spleen, and red marrow after cycle 1 and cycle 2. The AD/A admin presented in the table were calculated with personalized organ masses

Patients	Absorbed dose per administered activity (mGy/MBq)							
	Liver		Kidneys		Spleen		Red marrow	
	Cycle 1	Cycle 2	Cycle 1	Cycle 2	Cycle 1	Cycle 2	Cycle 1	Cycle 2
1	NA	0.29	NA	0.42	NA	0.89	NA	0.07
2	1.92	†	0.28	†	0.59	†	0.07	†
3	0.17	0.17	0.47	0.49	0.88	0.59	NA	NA
4	0.46	0.32	0.39	0.46	0.42	0.58	0.02	0.03
5	0.28	0.23	0.34	0.35	0.49	0.48	0.04	0.01
6	0.15	0.19	0.27	0.36	0.45	0.76	0.03	0.02
7	2.26	1.22	0.40	0.49	0.55	0.70	0.03	0.02
8	0.13	0.12	0.44	0.54	0.52	0.55	0.04	0.04
9	0.33	0.31	0.34	0.32	0.58	0.56	0.07	0.05
10	0.55	0.59	0.45	0.50	0.57	0.75	0.03	0.04
11	0.64	1.03	0.50	0.91	0.70	0.76	NA	NA
12	0.22	0.22	0.35	0.37	0.56	0.51	0.06	0.09

*NA* not available, † patient dead

Comparison of the AD/A admin values in ten patients (patients 3 to 12) after the first and second cycle of ^177^Lu-[DOTA0, Tyr3]-octreotate (Fig. 4) highlighted some minor between-dose differences in all four OARs (*p* = 0.0563 for kidneys, *p* = 0.3414 for spleen, *p* = 0.5106 for liver, and *p* = 0.7220 for red marrow). The ${\overline{\Delta}}_{AD/\mathrm{A}\ \mathrm{admin}}$ was 14.4% (range 0–41.4%) for liver, 12.5% (range 2–41.4%) for kidneys, 14.4% (range 2.2–36.4%) for spleen, and 32.8% (range 0–80.9%) for red marrow. The highest between-dose differences were observed in liver, in patient 7 (2.26 and 1.22 mGy/MBq for cycle 1 and cycle 2; Δ_*AD*/A admin *liver*, *P*7_ of 42.3%) and patient 11 (0.64 and 1.03 mGy/MBq; Δ_*AD*/A admin *liver*, *P*7_ of 33%).

**Fig. 5:**
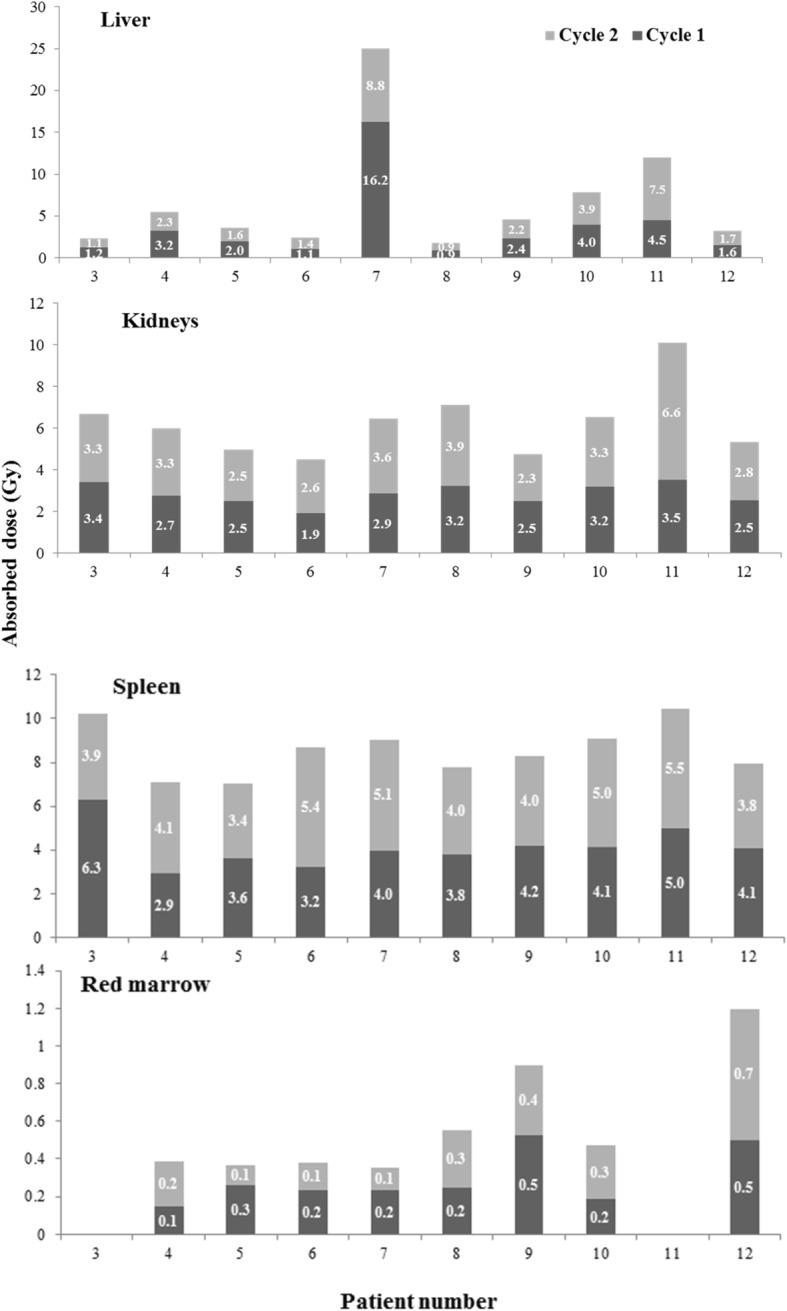
Absorbed doses in Gy in liver, kidneys, spleen, and red marrow after cycle 1 and after cycle 2. As our dosimetry methodology was based on L2-L3-L4 red marrow delineation, it could not be used for patients 3 and 11 who had tumors in contact with L2, L3, L4 and L2, L3 or L4 vertebral metastases

Similarly, when comparing the absorbed dose (in Gy) between cycles, intra-patient differences were observed especially in organs very close to or including tumor cells or metastases (Fig. 5). This was the case for liver in patient 7 (diffused liver metastases) and for red marrow in patients 3 and 11 (tumors in contact with L2–L4 and L2, L3, or L4 vertebral metastases) (data not shown). For organs without metastases or distant from the tumor, no significant between-cycle difference was observed (*p* = 0.0809, *p* = 0.4604, *p* = 0.5083, and *p* = 0.8720 for kidneys, spleen, liver, and red marrow respectively), except for kidneys in patient 11 due to an obstruction of the double pigtail stent before the second injection.

##### Mean absorbed dose per unit of administered activity to organs for all patients

**Fig. 6:**
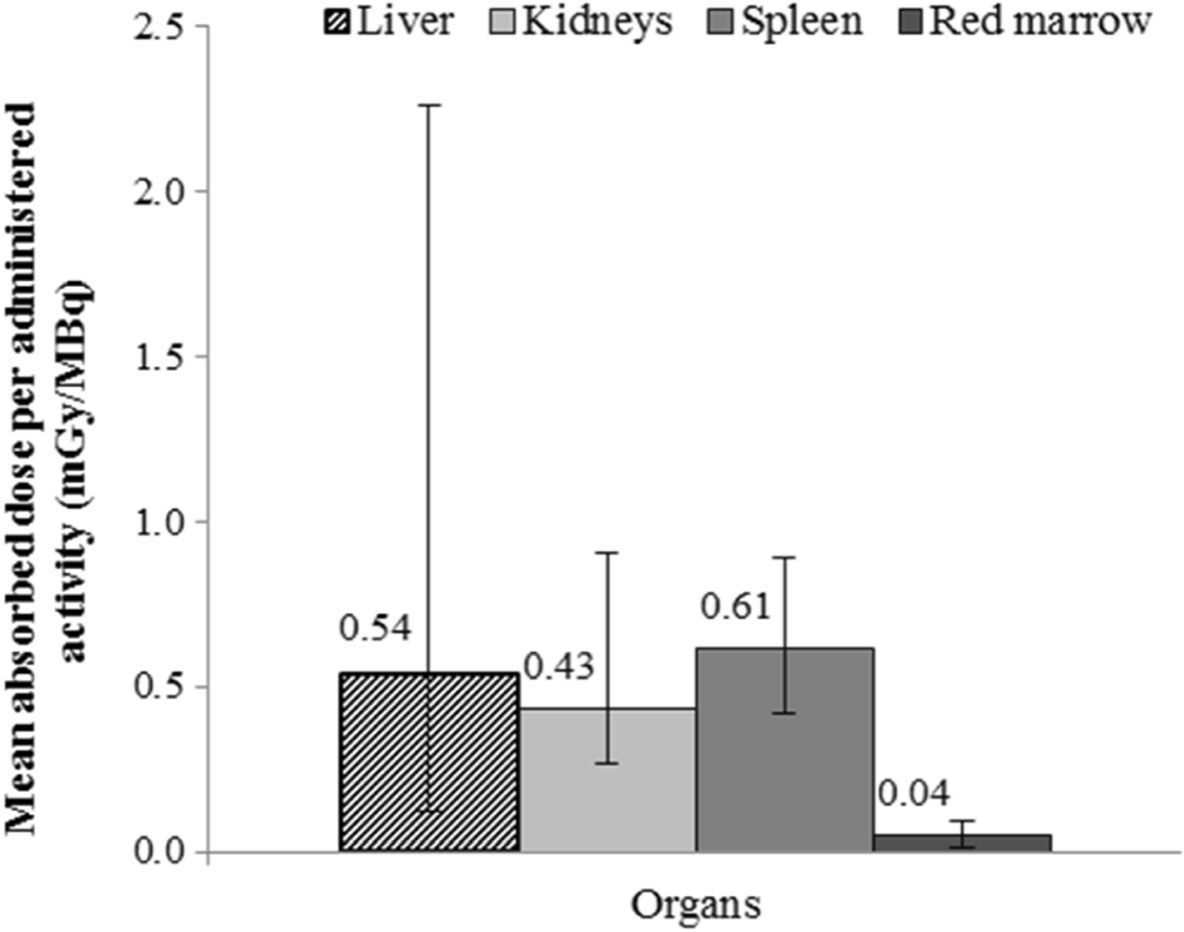
Mean absorbed dose to organs per administered activity in mGy/MBq for all patients. The error bars show the range of the AD/A admin values for cycle 1 and 2

The mean organ AD/A admin for all patients and the first two cycles were 0.54 ± 0.58 mGy/MBq for liver, 0.43 ± 0.13 mGy/MBq for kidneys, 0.61 ± 0.13 mGy/MBq for spleen, and 0.04 ± 0.02 mGy/MBq red marrow (Fig. 6).

## Discussion

This study reports our experience with dosimetry in patients with NET after LUTATHERA® treatment, and the absorbed doses to the kidneys, liver, spleen, and red marrow, using SPECT/CT data and the commercial workstation Xeleris®.

Activity quantification from the SPECT images is a crucial step. Therefore, calibration of the gamma camera is a prerequisite procedure in targeted radionuclide dosimetry [24, 25]. In our study, we reproduced the calibration method described in the MIRD pamphlet n°26 [18] using a large water cylinder containing a well-calibrated source of ^177^Lu, a phantom often employed in clinical studies [15, 26, 27]. We used the SPECT sensitivity factor according to the study by Frey et al. [13], and to a recent multi-center evaluation on phantoms [28]. The obtained recovery coefficient results and reconstruction parameters were in agreement with those of recently published studies [29, 30]. For patient dosimetry, SPECT/CT image acquisition at different time points was chosen to avoid the overlap with local high activity areas near the kidneys that could give an overestimation of the kidney activity, as described by Garkavij et al. [31]*.*

In addition, to better evaluate the accuracy of the AD/A admin values, especially for red marrow, we used another more suitable phantom to model the in vivo conditions in terms of organ volume and activity concentration. With this phantom, the recovery coefficient obtained for the L2–L4 model of 80 ml was in the same order of magnitude as the value obtained for a 16 ml sphere without background activity, possibly due to the high concentration gradient between the L2–L4 model and kidney model.

To evaluate the quantification accuracy, other calibration methods with more complex phantoms have been described. For example, Gnesin et al. [32] used an anthropomorphic phantom (Kyoto liver/kidney phantom) including kidneys and liver with spheres of 20, 30, and 40 mm in diameter to mimic lesions. Recently, Tran-Gia et al. [33] presented a 3D-printed two-compartment kidney phantom. A similar phantom for red marrow quantification and dosimetry remains a real challenge. According to Lassman et al. [16], the joint European project on MRT dosimetry is working on a standardization of SPECT/CT calibration procedures that will be available by the end of 2019. Given the diversity of dosimetry data published using different methodologies, Gear et al. [34] recently proposed a practical guide to express the accuracy of the dosimetry results by determining the uncertainties at each step of the dosimetry chain.

After this preliminary calibration step using phantoms that was necessary to obtain accurate quantification of the activity, we implemented the method in the clinic. We observed considerable differences of AD/A admin values for all the OARs, using the personalized and the standard organ masses. The personalized organ masses usually led to lower kidneys and spleen AD/A admin values, as previously reported by Kupitz et al. [20], and higher red marrow AD/A admin values in most patients. Thereby a correction for the individual organ masses was applied.

Based on the report [5] by Eberlein et al. that summarizes data on the red marrow, kidneys, and tumor absorbed doses from various studies, the range of the kidney AD/A admin values in our patients was in concordance with the values reported by Sandström et al. [27], Gupta et al. [35], and Heikkonen et al. [26]. The mean liver AD/A admin value was slightly higher than what previously published, possibly due to the presence of liver metastases in patient 7. Indeed, the mean and the range of liver AD/A admin values were in accordance with the literature after exclusion of this patient [35]. The mean red marrow AD/A admin was similar to published data. However, in our study, the dosimetry methodology was not based on blood sampling, but on L2–L4 red marrow delineation, and could not be applied to patient 3 and 11 who had tumors in contact with L2–L4 vertebrae and L2, L3, or L4 vertebral metastases. With our methodology, a high residence time for this volume of interest was extrapolated to the total red marrow, but in these two patients it was not representative. When excluding the data for these two patients from the analysis, the red marrow AD/A admin value was concordant with previously published data. Ferrer et al. [36] showed that although blood-based approaches are easier to implement, an imaging-based method better predicts the hematological toxicity. They reported that the approach based on L2–L4 vertebra imaging seemed to provide the best relation between absorbed dose to red marrow and platelet toxicity in radioimmunotherapy with ^90^Y-epratuzumab tetraxetan. Blakkisrud et al. evaluated the correlation between absorbed dose and hematologic toxicity in patients treated with ^177^Lu-lilotomab satetraxetan [37], and concluded that hematologic toxicity can be predicted by calculating the red marrow absorbed dose from SPECT/CT images. Recently, Beykan et al [38] estimated that during PRRT with ^177^Lu-DOTATATE, the blood-based absorbed dose to bone marrow was three times lower than the imaging-based absorbed dose, advocating the use of imaging-based dosimetry.

In our study, intra-patient differences of absorbed dose between cycle 1 and 2 were observed, especially for organs very close to, or including tumor cells or metastases. This could be explained by a lower uptake in the treated lesions between cycles. This suggests that the activity kinetic could be similar in organs containing or in contact with tumors and in the NET, and could explain the difference in absorbed dose between cycles.

For the kidneys, the reproducibility between absorbed dose after cycle 1 and 2 appeared at the limit of the significance. Indeed, intra-patient variability of the absorbed dose between cycles was previously reported by Sundlöv et al [11], possibly due to intra-patient pharmacokinetic variations between cycles. Particularly, variations of blood pressure and body hydration, and thus renal perfusion, could affect the residence time and the absorbed dose in kidneys.

Previous studies [35, 39] have shown that also in healthy tissues, the time-activity curve shape can differ considerably among patients and in the same patient between treatment cycles. The impact of kinetics and image timing on the absorbed dose to organs is crucial [40]. Consequently, our dosimetric results are clearly restricted to the use of the Dosimetry Toolkit® software because the time activity curves for each segmented organ were fitted automatically by a mono-exponential function to calculate the residence times.

Although other analytic fit functions, such as bi- or tri-exponential functions, are known to better fit the time-activity curves [40, 41], these options were not implemented in the software. Moreover, the Dosimetry Toolkit® software has a limited set of segmentation tools, is not user-friendly, and not optimal. Another major drawback is that this software cannot save the images and volumes of interest in Dicom format from the beginning to the end of the process. For example, it is not possible to change organ delineation after the process. In addition, the Dosimetry Toolkit® software is associated with a specific gamma camera by a license system. The software only accepts images obtained with a gamma camera with a known license number. Consequently, imaging data obtained with other gamma cameras cannot be used. However, new solutions are commercially available with user-friendly tools for segmentation, including those commonly used for EBRT (such as Boolean operators, easy manual, semi-automatic, automatic segmentation options…) and for nuclear medicine. These software programs also propose a wide choice of interpolation methods to better fit the obtained time-activity data.

From a practical point of view, as reported in the recent review by Huizing et al [42], dosimetry in PRRT is not routinely performed due to implementation difficulties, the time-consuming, non-standard dosimetry approach, and the lack of evidence in literature. Our study shows that routine patient dosimetry is achievable in a nuclear medicine department, if there is a strong medical willingness and awareness on how to perform patient dosimetry. As discussed by Flux et al. [43], a solid collaboration is needed between nuclear medicine physicians, medical physicists, and nuclear medicine technologists to implement dosimetry and provide the best patient care. Dosimetry of organs at risk and tumors brings real benefits for patients; however, health professionals must be aware that it is time-consuming using a gamma camera, and must anticipate the practical constraints before its routine implementation. In our clinical dosimetry protocol, each SPECT/CT acquisition by the gamma camera required about 25 min (i.e., an additional acquisition time of 1.25 h for the dosimetry of one patient after one treatment cycle). The registration, segmentation, and dosimetry steps also are time-consuming (around 1.5 h for one dosimetry). Despite the longer exam time, all imaging acquisitions were well received by our patients.

According to Hänscheid et al. [44], besides the additional burden for patients, this may also increase the complexity of data collection and analysis. Therefore, they proposed a simplified PRRT dosimetry method using a theoretical approach based on one single measurement of the activity retention at 96 h post-drug administration. Recently Sundlöv et al [45] evaluated in 22 patients (119 treatment cycles) a treatment planning schema that gives reliable dosimetric results with simplified acquisitions. They confirmed previous conclusions with the use of one SPECT/CT at 96 h after each treatment cycle. However, these results have to be confirmed with a large patient cohort.

## Conclusions

This personalized dosimetric study in patients with NET who underwent PPRT with ^177^Lu-DOTATATE highlights the important inter-patient/intra-patient variations and consequently the need of individualized dosimetry. Additional studies are needed to estimate the absorbed doses to tumors and to perform dose-response correlations.

It also shows that implementing patient dosimetry in the clinical practice during PPRT with ^177^Lu-DOTATATE is feasible in a nuclear medicine department.

## Abbreviations

AD/A admin: Absorbed dose per administered activity
EBRT: External beam radiotherapy
MEGP: Medium-energy general purpose
MIRD: Medical internal radiation dose
NET: Neuroendocrine tumor
OAR: Organ at risk
OSEM: Ordered subset expectation maximization
PRRT: Peptide receptor radionuclide therapy
SPECT/CT: Single-photon emission computed tomography/computed tomography
WB: Whole body
